# COVID-19 and cytokine storm syndrome: can what we know about interleukin-6 in ovarian cancer be applied?

**DOI:** 10.1186/s13048-021-00772-6

**Published:** 2021-02-08

**Authors:** Antonio Macciò, Sara Oppi, Clelia Madeddu

**Affiliations:** 1 Department of Gynecologic Oncology, Businco Hospital, “Azienda di Rilievo Nazionale ad Alta Specializzazione G. Brotzu”, Via Jenner, 09100 Cagliari, Italy; 2 Hematology and Transplant Center, Businco Hospital, “Azienda di Rilievo Nazionale ad Alta Specializzazione G. Brotzu”, Cagliari, Italy; 3 Department of Medical Sciences and Public Health, University of Cagliari, Cagliari, Italy

**Keywords:** Ovarian cancer, COVID-19, Immunopathology, Resistance, Tolerance, Interleukin-6, Oxidative stress, Corticosteroids

## Abstract

Improving early diagnosis along with timely and effective treatment of COVID-19 are urgently needed. However, at present, the mechanisms underlying disease spread and development, defined prognosis, and immune status of patients with COVID-19 remain to be determined. Patients with severe disease state exhibit a hyperinflammatory response associated with cytokine storm syndrome, hypercoagulability, and depressed cell-mediated immunity. These clinical manifestations, sharing similar pathogenesis, have been well-studied in patients with advanced ovarian cancer. The present review suggests treatment approaches for COVID-19 based on strategies used against ovarian cancer, which shares similar immunopathology and associated coagulation disorders.

The chronicization of the hyperinflammatory cytokine storm in patients with severe COVID-19 highlights a defective resistance phase that leads to aspecific chronic inflammation, associated with oxidative stress, which impairs specific T-cell response, induces tissue and endothelial damage, and thrombosis associated with systemic effects that lead to severe multi-organ failure and death. These events are similar to those observed in advanced ovarian cancer which share similar pathogenesis mediated primarily by Interleukin-6, which is, as well demonstrated in ovarian cancer, the key cytokine driving the immunopathology, related systemic symptoms, and patient prognosis.

Consistent with findings in other disease models with similar immunopathology, such as advanced ovarian cancer, treatment of severe COVID-19 infection should target inflammation, oxidative stress, coagulation disorders, and immunodepression to improve patient outcome. Correctly identifying disease stages, based on available laboratory data, and developing a specific protocol for each phase is essential for effective treatment.

## Introduction

At the end of 2019, an outbreak of atypical pneumonia was reported in Wuhan, China. Severe acute respiratory syndrome coronavirus 2 (SARS-CoV-2) was identified as the pathogen underlying this novel disease, later referred to as Corona Virus Disease 2019 (COVID-19). Since its first reported case, the infection has spread to other regions in China and other countries, and, despite aggressive containment efforts, the number of affected individuals is rising worldwide [1]. Furthermore, the fatality rate is very high, dominated mostly by the elderly and patients with comorbidities [2–4]. Patients with COVID-19 primarily present with fever, myalgia or fatigue, and dry cough. However, they may also develop dyspnea and hypoxemia within 1 week of disease onset, which may rapidly progress into acute respiratory distress syndrome (ARDS) or organ failure [4, 5].

COVID-19 develops through several stages, from mild to critically severe, thereby justifying an individualized response for each patient [6]. During the early disease stages, symptoms of severe acute respiratory infection can occur, with some patients rapidly develop ARDS and other serious complications, ultimately causing multiple organ failure. Therefore, early diagnosis and timely treatment is essential to improve patient outcome. At present, the mechanisms underlying disease spread and development, appropriate methods to determine prognosis, and immune status of COVID-19 patients remain under investigation.

The manifestation of severe forms of COVID-19 in a proportion of patients, highlights the inability of the immune system to counteract the viral infection. These patients develop hyperinflammation characterized by high levels of pro-inflammatory cytokines, primarily Interleukin (IL)-6, and other acute- phase proteins, including C-reactive protein (CRP), fibrinogen, and ferritin. They also exhibit lymphopenia, especially reduced CD3+ and CD4+ T- cell counts, and immunodepression, as well as high levels of D-dimer and fibrinogen degradation products [7–11]. Zhou et al. [9], was among the first to assessed temporal changes of laboratory circulating markers in hospitalized patients with COVID-19 from illness onset and demonstrated that patients who did not survive had significantly higher and progressively increasing levels of IL-6, serum ferritin, and D-dimer beginning on days 7 to 10 compared with those who survived. Patients who did not survive also had progressive neutrophilia with concomitant lymphocytopenia, in more severe cases. These parameters define the neutrophil/lymphocyte ratio (NLR), which provides a consolidated prognostic parameter for other diseases [12, 13], including ovarian cancer [14–17]. Noteworthy, during hospitalization evaluation, fibrinogen and antithrombin levels were significantly low in non-survivors [18]. High IL-6, CRP, ferritin, and D-dimer levels, as well as lymphopenia, are predictors of fatality [7, 9, 11]. Furthermore, advanced age (≥ 65 years), high fever (≥ 39 °C), comorbidities (e.g., hypertension or diabetes), and elevated end organ-related indices (e.g., aspartate aminotransferase, urea, and lactate dehydrogenase levels) are also associated with higher risk of developing ARDS [7]. A comparison of these hematological parameters in the mild and severe infection groups showed significant differences primarily in IL-6 and CRP levels, as well as coagulation function-related indicators (fibrinogen, d-dimer and time-to-thromboplastin) [7]. Specifically, IL-6 levels ≥24.3 pg/mL and D-dimer > 0.28 μg/L were closely correlated with the occurrence of severe COVID-19, and with their combined detection provided the highest specificity and sensitivity for early prediction of disease severity and death. Hence, IL-6 has been defined as the emblematic cytokine for this severe infection [9]. Importantly, inflammatory indexes (CRP) and NLR have been shown to be reliable parameters for treatment monitoring [19, 20]. A study on 61 consecutive patients with COVID-19 infection treated at Beijing Ditan Hospital from January 13 to 31, 2020, demonstrated that NLR was a meaningful parameter for prognosis as well as for risk stratification and management, with a NLR cut-off value of 3.13 providing high sensitivity and specificity for the prediction of disease severity [21]. Accordingly, the authors suggested that patients that are > 50 years of age with an NLR > 3.13 may require intensive care, whereas those with an NLR of < 3.13 and age < 50 years could isolate at home or be treated in a community hospital setting. These indications can alleviate the current pressure on global health community due to insufficient medical resources.

These observations indicate that COVID-19 patients with severe disease exhibit a hyperinflammatory response, primarily driven by IL-6, associated with cytokine storm syndrome, blood hypercoagulability, and depressed cell-mediated immunity [22, 23]. At this regard, in 1998 we had demonstrated that high levels of pro-inflammatory cytokines such as IL-6, tumor necrosis factor (TNF)-α, IL-1, and CRP are correlated with impaired T-cell responses in women with advanced epithelial ovarian cancer [24]. We have also previously demonstrated that increased levels of macrophage-derived pro-inflammatory cytokines, primarily IL-6, during evolution of ovarian cancer, are associated with the onset of systemic symptoms, including anemia, anorexia, and fatigue, as well as peripheral, cardiac, and respiratory muscle wasting [25–29]. In 2005, for the first time, we reported that, in a population of advanced ovarian cancer assessed at diagnosis prior to any antineoplastic treatment, the severity of cancer-related anemia was associated with a status of chronic inflammation characterized by high levels of IL-6, CRP, and fibrinogen [28]. Additionally, hemoglobin was significantly positively correlated with the nutritional status, defined by leptin values, and inversely correlated with high level of oxidative stress, characterized by elevated reactive oxygen species (ROS) and reduced antioxidant enzymes [28]. In a larger cohort of patients with advanced cancer at diagnosis before antineoplastic treatment, where patients with ovarian cancer showed the most severe anemia, low hemoglobin levels were associated with high hepcidin and ferritin levels, which were positively correlated with IL-6 [30]. Consistently, a recent meta-analysis of iron metabolism in anemia associated with COVID-19 showed that patients with severe disease had lower hemoglobin and higher ferritin, and the mean ferritin levels significantly differed between survivors and non-survivors [31].

Additionally, in a landmark study of paraneoplastic thrombocytosis in patients with epithelial ovarian cancer, Stone et al. [32] conducted parallel clinical and experimental analyses and found that advanced disease was associated with thrombocytosis, which was significantly correlated with the levels of IL-6 and thrombopoietin and associated with a significantly shorter progression-free and overall survival. Moreover, the authors also showed that both in ovarian cancer animal model and in patients with ovarian cancer, blocking IL-6 production with small interfering RNA or siltuximab, an anti-IL-6 antibody, respectively, normalized platelet counts. Since then, a paracrine circuit involving IL-6, which increases the levels of fibrinogen and thromboplastin thereby causing thrombocytosis, has been found to be associated with increased thrombotic risk in patients with ovarian cancer and other neoplasms. Notably, among the different tumors, ovarian cancer has the highest incidence of tumor-associated thrombocytosis and the strongest experimental and clinical evidence on the role of inflammation (particularly IL-6) in the development of this paraneoplastic condition [33–35].

The above observations illustrate how COVID-19 follows the paradigm of chronic inflammation development attributed to the failure of the resistance phase, consequent to the inability of the adaptive immune system to eliminate the pathogen, together with simultaneous activation of deleterious compensatory mechanisms, similar to those of the tolerance phase [36]. Thus, our knowledge of these processes could help to develop a more effective rationale treatment strategy for COVID-19, rather than an empirical approach. Indeed, the initial phase of the COVID-19 cluster cases was not well understood, which could have caused errors in patients’ evaluation and treatment, particularly those with severe infection. Thus, the present review further expands this field by suggesting a rational treatment approaches based on evidence from studies on other diseases with similar immunopathology, such as advanced ovarian cancer. Similarly, in a recent mini-review, Turnquist et al. [37], used lung cancer to illustrate the similarities between the cytokine storm of COVID 19 and that observed in cancer. However, the available data and the long-lasting research on similar immunopathology is even more evident in ovarian cancer.

### The phases of avoidance, resistance and tolerance during the COVID-19 infection

Humans are readily infected by various viruses, however in most cases, resolve the infection with or without tissue damage. The host can protect itself from infectious disease using three distinct strategies: Avoidance, resistance, and tolerance [38]. Avoidance involves reducing the risk of exposure to infectious agents by avoiding contact with those possibly infected; this remains the most important preventive measure. Hence, public health measures to contain the virus by minimizing social contact have the essential role of avoiding widespread infection. Alternatively, resistance and tolerance rely on the host response, since they are a function of pathogen burden and immunogenicity, and on the ability of the immune system to mount an efficient response [39].

#### Resistance phase

Resistance reduces pathogen burden once the infection is established. During this phase, the immune system recognizes the antigenic diversity of the pathogen and, to the extent that the immune system is effective, eliminates it. In this stage, the activities of macrophages, dendritic cells, natural killer cells, T and B lymphocytes, and, in certain cases, neutrophils, are determinantal. These cells, in turn, release a range of molecules that can induce tissue damage and organ malfunction, including interferons (IFNs), cytokines, cationic proteins, lipid mediators, metalloproteinases, and ROS [40]. ROS accumulated in the mitochondria strongly contributes to tissue damage and endothelial dysfunction [41, 42], thereby activating the pathogenetic mechanisms of thrombosis. Indeed, alterations of the vascular endothelium results in altered generation of thrombin, both systemically and locally in the lungs of patients with severe pneumonia, resulting in the deposition of fibrin with subsequent tissue damage and coagulation pathology [43]. Importantly, adaptive immune effector cells can induce tissue damage. For instance, CD8+ T cells can directly destroy virus-infected cells, however, in some non-cytopathic viral infections, such as those caused by HCV and HBV, the destruction of infected cells by CD8+ T effectors is also the primary cause of liver damage [44, 45]. Similarly, antibodies binding to the antigens presented on infected cell surface influence the activation of complement cascades which induce an inflammatory reaction, thereby contributing to tissue damage [40].

During COVID-19 infection, the primary site of injury is the lung [46]. Experimental analysis showed that SARS-CoV-2, the etiologic agent of COVID-19, uses angiotensin-converting enzyme 2 (ACE2), which is primarily expressed in a small subset of cells in the lung called type 2 alveolar cells, as a cell receptor to gain entry to the cell [47]. Additionally, it has been reported that SARS-CoV-2 directly infect alveolar macrophages; a key feature in SARS-CoV viruses-mediated pathogenesis [48]. Therefore, it is within the lungs that the host-pathogen clash occurs.

The initial phase of COVID-19 infection can be successfully controlled in most individuals. However, in some, it exhibits intrinsic properties making any attempt to control the virus result in tissue damage. The greatest tissue damage occurs in individuals with predisposing genetic, or acquired conditions, involving one or more components of the innate or adaptive defense system, or in older patients [36, 40].

**Fig. 1:**
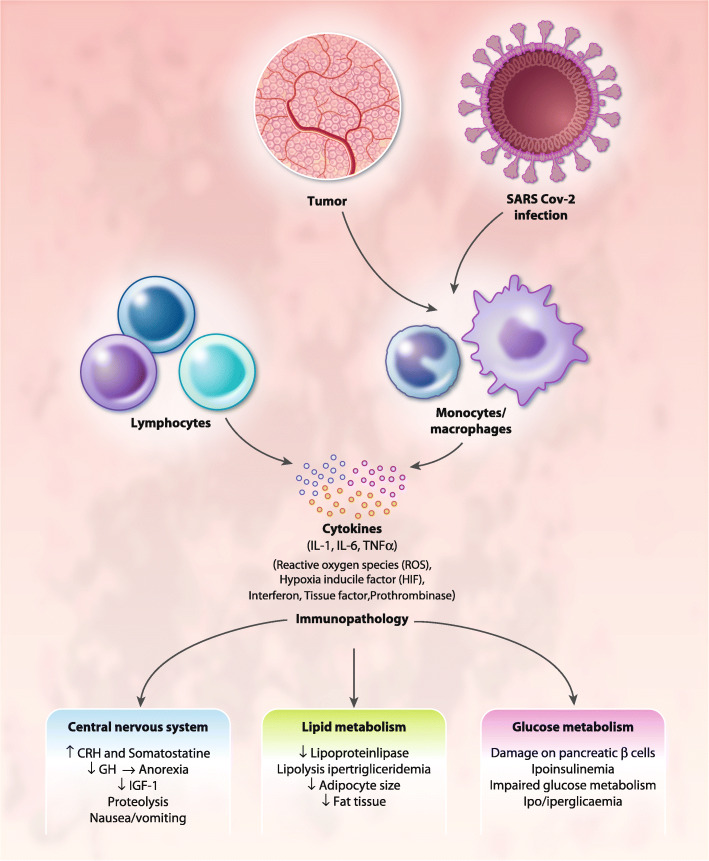
Mechanisms of immunopathology in ovarian cancer and COVID19. Both in ovarian cancer and COVID-19 activated and/or infected macrophages induce an inflammatory response with prolonged and excessive production of proinflammatory cytokines, Interferon, Reactive Oxygen Species, and procoagulant factors that results in immunopathological changes, thus leading to symptoms associated with sickness behavior, including anorexia and specific changes in energy metabolism. Abbreviations: IL, Interleukin; TNF, Tu*mor Necr*osis Factor; CRH, corticotrophin releasing hormone; GH, growth hormone; IGF, Insulin growth factor

Such tissue damage cause widespread necrosis, which induces an additional strong nonspecific inflammatory response, fundamentally driven by macrophages and their cytokines; hence, macrophages are the key cell type driving immunity during all phases of COVID-19 infection. Alveolar macrophages may be infected by the virus, which propagates inside the immune cells thereby escaping early induction of IFN response, which is involved in the initiation of the innate immune response. Meanwhile, infected macrophages show increased and dysregulated production of, among others, chemokines and pro-inflammatory cytokines, as well as procoagulant prothrombinase [49]. Moreover, alveolar macrophages activated by viral pathogen-associated molecular patterns and by cytokines/chemokines released by infected cells (including infected macrophages), represent the first line defense against viral infection at entry site and their behavior and polarization influence disease outcomes [50]. Macrophages during the early phases detect the infection and respond by inducing IFN release, which limits viral replication. Furthermore, through the release of IL-1, IL-6, and TNF-α, macrophages modulate the activity of CD4^+^ T cells by inducing the production of IL-2; while also inducing the expression of the IL-2 receptor, particularly on cytotoxic CD8^+^ T cells [40]. However, prolonged and excessive production of pro-inflammatory cytokines and ROS by activated macrophages induce an inflammatory response that results in symptoms associated with sickness behavior [49] (Fig. 1). This is also observed in cancer [51]; in particular, in ovarian cancer, the study of tumor microenvironment in ascites offers the optimal condition to assess the relationship between the tumor and the immune system [52–54]. Indeed, the first study that demonstrated the peculiar metabolic changes of tumor microenvironment was carried out by Warburg in monocyte from peritoneal effusion induced by an abdominal implanted tumor (similarly to ovarian cancer) [55]. Hence, the role of tumor-associated macrophages with their metabolic changes (Warburg effect) and consequent polarization in promoting tumor-associated inflammation as well as tumor progression (invasiveness and metastatic potential) and immune response modulation have been extensively studied in ovarian cancer both in experimental models and ex vivo [51, 56–62]. In this context, we demonstrated, using the ascites of patients with advanced serous papillifery ovarian cancer, a prevalence of M1 cells and an association between M1 macrophage polarization and high levels of IL-6, hepcidin, and ROS production [63]. In the same study, we also demonstrated that IL-6 is key to M1 macrophage polarization, the synthesis of hepcidin and, consequently, the elevated circulating levels of ferritin, and that the anti-IL-6 monoclonal antibody, tocilizumab, effectively inhibited M1 polarization, as well as hepcidin and IL-6 production [63]. We have identified, as a representative case among patients with COVID-19 admitted at the COVID hospital of Cagliari started from March 2020, a 61 years-old patient without comorbidities, affected by a worsening respiratory syndrome and pneumonia from COVID-19, and evaluated the phenotype of peripheral blood macrophages [63, 64]. We found a prevalence of M1 polarized macrophage with a M1/M2 ratio of 3.1, which was parallel with our previous findings in advanced ovarian cancer patients [63, 64], associated with high circulating levels of CRP, IL-6, and ferritin, as well as lymphopenia. Similarly, in 2018, Thorsson, while determining the immune phenotype associated to several types of cancer, showed that ovarian cancer had the highest M1/M2 ratio, among other cancers, associated with a prevalence of CD8+ cells and cytokines made by activated Th1 and Th17 cells, including IL-6 [65].

The novel coronavirus encodes proteins that inhibit IFN signaling and contribute to evasion of the adaptive immune system, which is closely associated with disease severity [66]. In other SARS-COV infections, the virus can evade the IFN signaling both at the stage of IFN induction via ubiquitination and degradation of the RNA sensor adaptor molecules or via inhibiting IRF3 nuclear translocation, as well as at the stage of IFN secretion, by decreasing STAT1 phosphorylation [67–69]. Next, active replication of SARS-COV likely induces a delayed type I IFN response resulting in compromised early viral control and increased influx of pro-inflammatory macrophages [66, 70]. At this point, all events associated with aspecific chronic inflammation become activated; therefore, the persistence of the virus and the activation of innate immunity result in excessive, prolonged nonspecific inflammation, which negatively impacts immunosurveillance [71]. These events are similar to those described ovarian cancer microenvironment and contribute to the well-known phenomena of immune editing and escape in cancer development [71, 72].

Hence, aspecific inflammation contributes to immunosuppression, thrombosis, and multi-organ failure, which, in turn, contribute to the development of severe COVID-19. Due to these events, the initial interaction between the pathogen and host transitions into a systemic disease characterized by specific symptoms with severe immunopathology [66]. Therefore, the immunopathology observed in the lungs of patients with COVID 19 is an unavoidable consequence associated with the immune defense. The degree of immunopathology is positively correlated with the magnitude and duration of the immune response; therefore, the optimal immune response can be defined as the balance between efficient clearance of the pathogen and an acceptable level of tissue damage [36].

#### Tolerance phase

The development of COVID-19, which overcomes the host defense mechanisms, results from ineffective immune resistance, which is followed by a chronic, aspecific inflammatory response exerted primarily by macrophages, that induces symptoms related to “sickness behavior” [36], including anorexia, fatigue, and anemia, which exemplifies the mechanism of tolerance. Tolerance is, therefore, a phase that occurs to limit the damage caused by chronic activation of the immune system following failed resistance [73].

Although the sickness behavior, typical of the tolerance phase, is assumed to be adaptive, it is not clear whether, or how, it benefits the infected host. For instance, anorexia and anemia may be attempts to preserve vital processes, however, they promote stress and damage in multiple tissues. Wang et al. has shown that anorexia is protective in bacterial sepsis while lethal in viral infections [74]. Although the resistance strategy is crucial for host protection, it has significant consequences. Indeed, the elimination of COVID-19 can be accompanied by excessive collateral tissue damage. Therefore, tolerance, the final defense strategy of the host, can result in further infection-induced damage [73]. These concepts are well known in advanced neoplastic disease, during which the transition from resistance to tolerance is responsible for anorexia/cachexia syndrome, which is the primary cause of death in several cancer patients; this syndrome has been the subject of our research for several years [29, 75–78]. The tolerance strategy may become activated when the inflammation-driven damage, caused by the immune system, equates that induced by the pathogen. Unlike resistance mechanisms, tolerance does not directly affect the pathogen burden, instead, it decreases host susceptibility to tissue damage or other fitness costs caused by the pathogen, or by the immune response [36, 73]. Tolerance is also associated with damage-limiting events that do not happen in the most severe cases of COVID-19. During the infection, multiple physiological processes are significantly altered with often unknown rationale. Depending on the infection, these alterations can include profound changes in metabolism as well as in hepatic, renal, and cardiovascular functions. In principle, these responses can be either beneficial, induced as part of a general defense program, or, as in the case of COVID-19 infection, unintended, detrimental, and unavoidable consequences of infection.

### The role of necrosis in the evolution of COVID-19 infection

During severe COVID-19 infection, tissue damage appears with widespread necrosis, which likely induces inflammatory signaling through the hypoxia-inducible factor (HIF)-1 pathway, thereby further activating macrophages and causing a switch in metabolic phenotype. Similarly, in cancer, HIF1α coordinates the production of inflammatory mediators, including cytokines and chemokines, as well as the production of cyclooxygenase 2 and prostaglandins. These factors recruit and activate various leukocytes, most notably monocytes and macrophages, resulting in higher production of inflammatory mediators and thereby generating a cancer-related inflammatory microenvironment [51]. In ovarian cancer, which is one among the tumors with the strongest expression of HIF [79], hypoxic microenvironment is associated with a metabolic switch, of both cancer and immune cells, characterized by increased glycolytic activity, which influences tumor-associated macrophages (TAM) activation, polarization, and functions [80, 81]. Indeed, ovarian cancer ascites has a very hypoxic microenvironment where the role of HIF-induced release of pro-inflammatory and immunosuppressive factors and hypoxia-induced metabolic changes in modulating immune cells and impair T-cell functions can be clearly demonstrated [53].

Hence, understanding the specific functional activities of macrophages, which are key cell types in the evolution of COVID-19, is fundamental for identifying optimal therapeutic strategies. HIF-1 could serve as a critical transcriptional regulator of adaptive immunity and inflammation in COVID-19, and its signaling and the induction of aerobic glycolysis occur during the activation of certain immune cells, most notably M1 polarized macrophages. The HIF-1 pathway induces a metabolic switch in these cells, allowing them to appropriately respond to significant changes in energy requirements that occur upon activation, and to adapt to hypoxic conditions in inflamed and necrotic tissues. Necrotic foci, caused by of inflammation induced in the lung during the infection, generate difficult microenvironments for lymphocytes, in which they must adapt to hypoxia, acidosis, and redox stress to survive. During this phase of infection, the activation of the immune system requires specific modifications to the energy metabolism mechanisms used by cells, wherein the macrophagic cytokine IL-6 plays a key role. From a metabolic point of view, hypoxia and inflammation are inherently linked. For instance, decreasing oxygen levels induces metabolic changes to sustain ATP production; meanwhile quiescent immune cells, which are metabolically inert, require substantial metabolic reprogramming upon activation to provide sufficient ATP for effector functions. HIF-1 signaling drives such metabolic shift in the activated immune system, particularly in macrophages [82]. This metabolic shift induces macrophages polarization to M1 phenotype, which are the primary producers of IL-6 [83, 84]. Further, IL-6, through the signal transducer and activator of transcription 3 (STAT3) activation, contributes to metabolic reprogramming which induces aerobic glycolysis and downregulates mitochondrial oxidative phosphorylation by promoting pyruvate dehydrogenase kinase activity, thus shifting glucose metabolism toward the synthesis of lactate with increased levels of lactate dehydrogenase [85]. Notably, in ovarian cancer, in vitro studies have illustrated how IL-6 could induce, through STAT-3, the upregulation of HIF [86] as well as overexpression of hexokinase and associated glycolytic pathway [87].

In summary, macrophages are essential elements during the initial phase of the immune response, due to their antiviral properties, capacity to synthesize IFN, and interactions with, and recruitment of, helper and cytotoxic T lymphocytes. However, their persistent activation leads to the impairment of effective T-cell responses by causing T-cell exhaustion, a condition where the lymphocytes, even when activated, are nonfunctional and subsequently undergo programmed cell death [71, 73], which may contribute to the observed lymphopenia in COVID-19. Recently, we have highlighted how blocking chronic inflammation, primarily driven by IL-6, may improve the efficacy of the currently available immunotherapy in cancer patients [71].

Recently, Wu et al. [7] showed that high IL-6 expression is one of the most important parameters indicative of severe COVID-19, which is associated with lymphopenia, a serious condition, and mortality. Similarly, we examined the effects of IL-6 in patients with advanced cancer, particularly ovarian cancer, and found that its deleterious effects must be counteracted at all stages of metastatic neoplastic disease [25–28, 63].

Indeed, in ovarian cancer, the constitutive production of IL-6 by cancer cells has been strongly demonstrated [88], as wells as IL-6 ability to promote tumor growth and progression through autocrine and paracrine actions [89, 90] and to cause specific immune and metabolic alteration that impact prognosis [91].

**Fig. 2:**
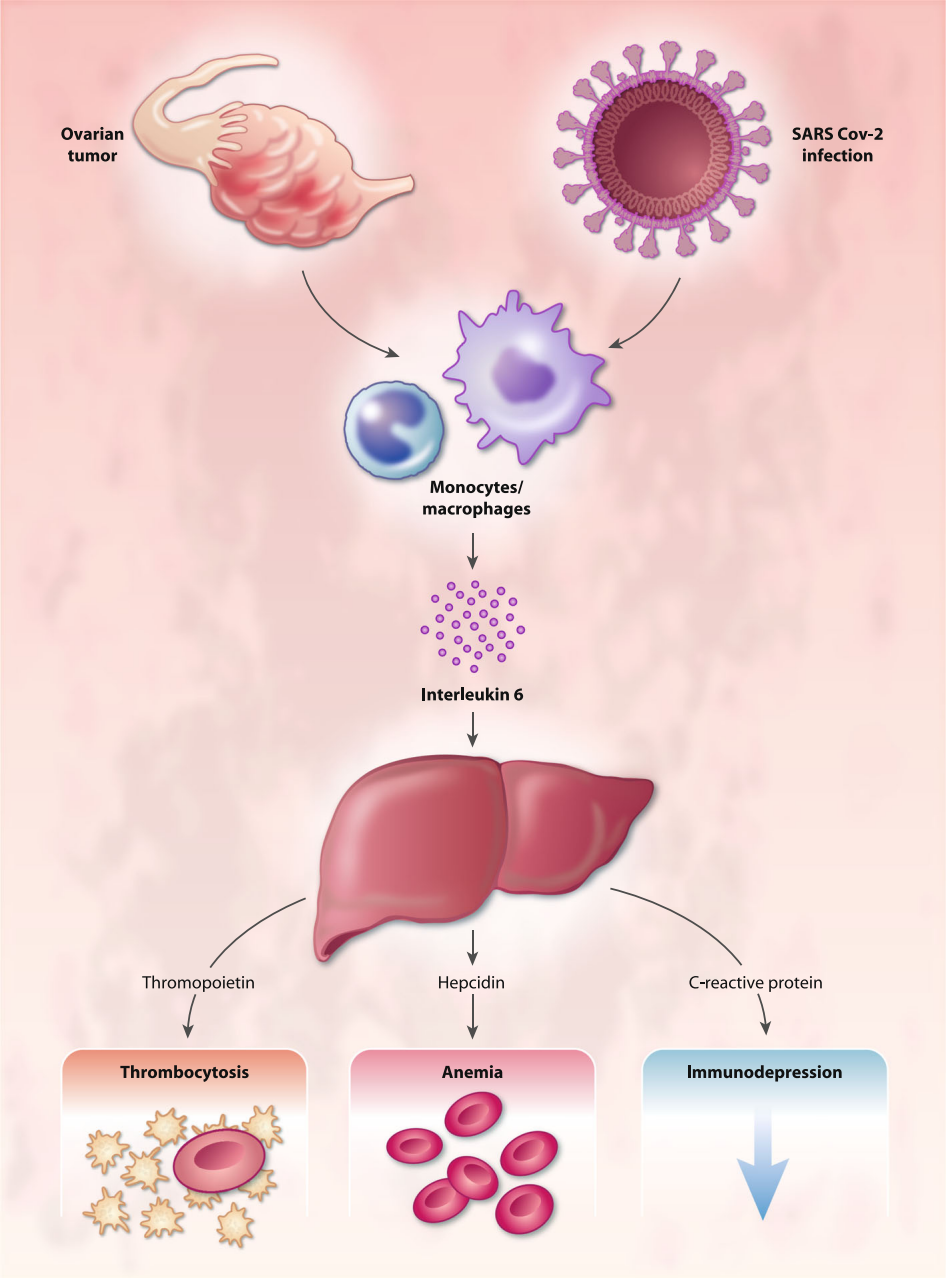
Role of IL-6 in immunopathology associated to ovarian cancer and COVID-19. IL-6 is one of the key cytokines driving the immunopathology caused by prolonged aspecific inflammation. IL-6 induces the transcription of several liver-specific genes in acute inflammatory states, such as CRP, thrombopoietin, and hepcidin thus contributing to associated immunodepression, anemia and thrombocytosis (with related hypercoagulability)

Also, in COVID-19, IL-6 is one of the key cytokines driving the immunopathology caused by the prolonged aspecific inflammation and correlated symptoms (Fig. 2).

### Interleukin-6 actions and immunopathology

IL-6, a 26-kDa glycopeptide encoded by a gene on chromosome 7, is produced by antigen-presenting cells such as dendritic cells, macrophages, and B cells. It is also produced by non-hematopoietic cells including keratinocytes, fibroblasts, epithelial cells, and neoplastic cells. IL-6 gene transcription is induced in normal tissues in response to stimuli including viral infection, bacterial endotoxin, lipopolysaccharide, as well as inflammatory cytokines and IFNs. IL-6 binds ligand-specific α-receptors (IL-6 receptor-α, also known as gp80) in either membrane-bound or soluble forms to activate signaling. This ligand receptor complex then binds universally expressed type I cytokine receptor IL-6 signal transducer/gp130 to activate three main pathways: STAT1/3, extracellular signal-regulated kinase and phosphatidylinositol-3-kinase (PI3K)/protein kinase B (AKT)/mammalian target of rapamycin (mTOR) pathways, of which, the Janus kinase (JAK)/STAT is the best characterized. Binding of gp130 activates associated JAKs, which then engage Src Homology 2 (SH2)-containing proteins, inclusive of STAT3. STAT3 phosphorylation leads to dimerization, nuclear translocation, DNA binding, and target gene regulation [26]. IL-6 is one of the major immunoregulatory cytokines present in the ovarian cancer microenvironment [92] while IL-6 antagonists may exert therapeutic effects against ovarian cancer [93].

IL-6 has pleiotropic actions that can affect virtually every organ. As widely demonstrated in ovarian cancer, IL-6 acts both locally, by conditioning the host immune response at the site of pathogen infection, and systemically, by altering energy metabolism, hematopoiesis, and nutritional status, as well as inducing severe endothelial damage, thereby negatively affecting patient prognosis [25, 26]. Through its central role in the hypothalamic–pituitary–adrenal axis, IL-6 contributes to sickness behavior response by causing mood disturbances and neurovegetative symptoms, including anorexia, fatigue, lethargy, malaise, difficulty concentrating, reduced activity, sleep impairment, and disinterest in activity [94]. In animal models of ovarian cancer, plasma and/or central nervous system (CNS) IL-6 was found to be associated with reduced locomotion and depression-like behaviors [95, 96]. Moreover, a significant association between plasma IL-6 and vegetative depression, disability, sleep disturbances, and fatigue has been demonstrated in patients with ovarian cancer at the time of surgery [97, 98]. Consistently, Schrepf et al. [99] has demonstrated in patients with ovarian cancer that normalization of IL-6 levels following antineoplastic treatment was associated with declined self-reported fatigue, vegetative depression, and disability.

Neurological symptoms are also strongly associated with increased levels of ROS, which are associated with high IL-6 levels. The ROS inflammatory pathway may propagate within the brain and activate key molecular pathways involved in neurocognitive decline and neurological alterations [100, 101]. IL-6 also induces the transcription of several liver-specific genes in acute inflammatory states (Fig. 2), such as CRP and other acute-phase proteins such as Fibrinogen, in a dose- and time-dependent manner [102]. Using an in vitro model of ovarian cancer, it has been demonstrated that IL-6 produced by TAM attributes to the elevation of liver-derived acute-phase proteins [103].

Acute-phase proteins, in turn, contribute to IL-6-related pathogenesis, including immunodepression. Moreover, hepatic IL-6 induces the synthesis of hepcidin, ultimately causing functional iron deficiency, chronic inflammation-associated anemia [28], and thrombopoietin, which induces thrombocytosis [32], as specifically demonstrated in ovarian cancer [32, 33]. Notably, the increased levels of fibrinogen, high platelet count (thrombocytosis), and endothelial damage in patients with COVID-19 are possibly the primary factors responsible for pathogenesis of thrombosis and disseminated intravascular coagulation, of which the increased levels of D-dimer and reduced platelet count are the most evident indices [23]. Specifically, the development of disseminated intravascular coagulation is associated with progression of certain severe cases of COVID-19 from ARDS to death [7]. These factors explain the strong temporal correlations in severe COVID-19 patients observed between increases in the levels of IL-6, CRP, and D-dimer, and with lymphopenia and the development of progressive organ failure.

IL-6 influences the effectiveness of the immune system in multiple ways. For instance, IL-6 can act as an activator, or an inhibitor, of T-cell responses, depending on the duration of its activity. Indeed, it initially participates in the activation of the immune response; however, its prolonged, chronic release ultimately contributes to immunosuppression. IL-6 can also suppress T-cell proliferation by inhibiting IL-2 synthesis, IL-2 receptor expression, and IL-2-dependent JAK/STAT signaling [26]. In fact, in patients with advanced ovarian cancer, we have demonstrated a correlation between high levels of IL-6 and the inability of lymphocytes to produce adequate level of IL-2 or express IL-2 receptor at physiological levels [24]. Noteworthy, a link between high levels of soluble IL-2 receptor and lymphopenia of COVID-19 has been reported [104].

**Fig. 3:**
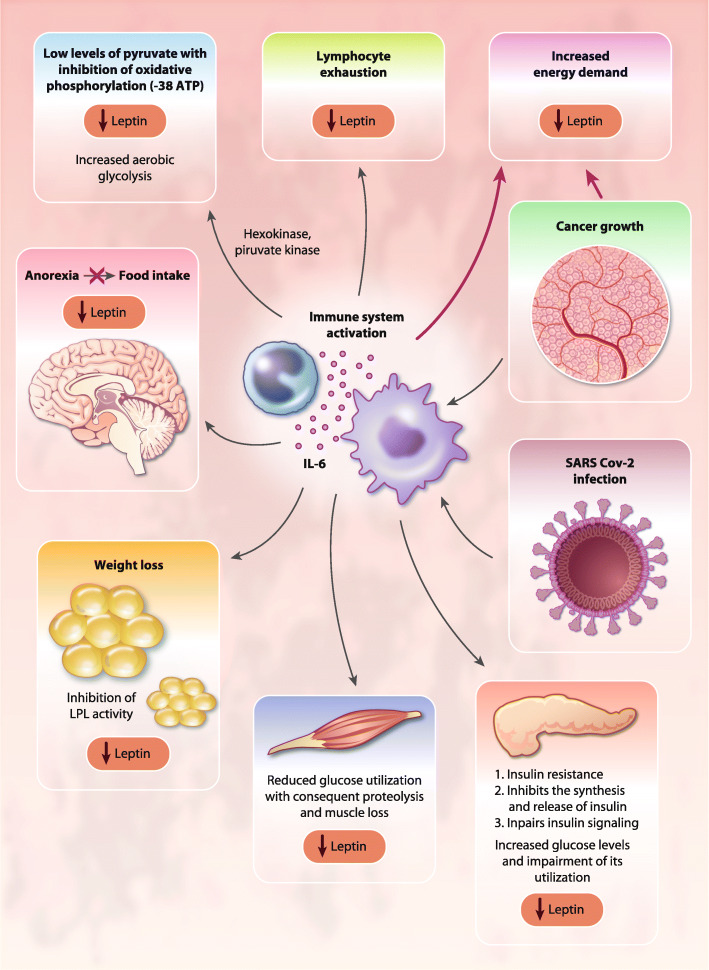
Systemic effects of IL-6 in ovarian cancer and COVID-19. IL-6 exerts systemic effects on energy metabolism, including the induction of peripheral muscle insulin resistance and the impairment of insulin signaling, which are associated with increased amino acid oxidation, negative energy balance, and muscle proteolysis. Abbreviations: IL, Interleukin; LPL, lipoprotein-lipase

Moreover, high concentrations of IL-6 may activate a STAT5-dependent negative feedback loop in CD4^+^ T cells, the primary producers of IL-2, thus altering the balance between STAT3-dependent CD4^+^ T cells, and STAT5-induced regulatory T cells [105]. Additionally, IL-6 may divert the immune system away from a Th1 response toward more immunosuppressive Th2 activity. Meanwhile, CRP, which is induced by IL-6, may interfere with the immunological mechanisms underlying the activity of IL-2 [106]. CRP is also involved in the binding of complement to cytotoxic CD3^+^ cells [107] and plays a key role in the inhibition of NK cell cytotoxicity [108]. Other negative effects exerted by IL-6 on the immune system result from its regulation of cellular glucose uptake, glycolysis, and iron trafficking. IL-6 disrupts energy and iron metabolism, leading to T-cell exhaustion, characterized by a progressive loss of T-cell function [71]. IL-6 can also exert direct inhibitory effects on the PI3K/Akt/ mTOR pathway, thereby inhibiting primary cellular energetic and anabolic processes [109]. Also, the systemic metabolic effects of IL-6 contribute to impaired T-cell energy pathways. Specifically, IL-6 signaling induces insulin resistance, alters protein, lipid, and fatty acid metabolism [85], and induces anemia [28] and anorexia [25], with consequent impairment of nutritional intake and utilization of energy substrates and microelements (e.g., glucose, iron, and zinc) that are fundamental for the activity of the main lymphocyte energy metabolic pathways (Fig. 3). To this regard, we had demonstrated in patients with advanced ovarian cancer a positive correlation between high IL-6 levels and impaired nutritional status, as evidenced by low levels of leptin [27]. Moreover, we have shown, in a large cohort of patients with advanced cancer evaluated at diagnosis before any antineoplastic treatment, where ovarian cancer patients showed the most severe anemia, that IL-6 correlated directly with the Glasgow Prognostic Score an inflammatory/nutritional index defined by the CRP/albumin ratio [30]. Overall, IL-6 signaling can cause an energy deficiency condition that represses T-cell receptor (TCR)-related signaling, IFN production, cytotoxicity, and T-cell motility, with detrimental effects on the immune response [71]. Thus, inflammatory immune response can deplete T cells and affecting the outcomes of patient with COVID-19 leaving them prone to secondary infection [110].

Among the clinical phenomena associated with IL-6-mediated immunopathology, muscle wasting, affecting respiratory muscles thereby contributing to respiratory dysfunctions, significantly impacts morbidity of patients with COVID19. IL-6 induces the loss of muscle mass [76]; for instance, IL-6 can exert systemic effects on energy metabolism, including the induction of peripheral muscle insulin resistance and the impairment of insulin signaling, which are associated with increased amino acid oxidation, negative energy balance, and muscle proteolysis (Fig. 3). Additionally, muscle loss is directly induced by IL-6-mediated activation of muscular STAT3/NF-kB signaling and ubiquitin-proteasome system-mediated proteolysis in skeletal muscle. Muscle wasting caused by activation of the IL-6/STAT3 signaling cascade also involves the inhibition of the PI3K/Akt/mTOR pathway, which induces muscle protein synthesis (anabolism) [111]. To this regard, Pin et al. [112] showed that ovarian cancer can serve as an optimal preclinical model for studying the role of IL-6 in inducing cancer-associated cachexia. The authors implanted ES-2 ovarian cancer cells intraperitoneally to simulate disseminated abdominal disease and malignant ascites formation and found severe weight loss with progressive muscle wasting via enhanced muscle protein catabolism associated with elevated tumor-derived IL-6 levels in plasma and ascites, elevated phospho-STAT3, reduced levels of phosphorylated AKT, and altered mitochondrial homeostasis and metabolism. Inhibition of the IL-6/STAT3 signaling restored muscle wasting [112]. The actions of IL-6 on muscle wasting are dose- and time-dependent. Indeed, recent data showed that patients with COVID-19 develop weight loss and cachexia that correlated with high levels of inflammatory parameters (CRP), impaired renal function status, and longer duration of COVID-19 disease [113]. Therefore, the prolonged exposure to high IL-6 in patients with severe COVID-19 may lead to serious muscle wasting, which significantly worsens lung functional capacity and, through the associated negative energy balance, contributes to organ failure, including respiratory failure, as similarly reported in other severe viral infectious diseases [114, 115].

### Role of oxidative stress in the evolution of COVID-19

Chronic inflammation and macrophage activation induce oxidative stress. We have previously demonstrated that high levels of IL-6, CRP, and other pro-inflammatory cytokines in patients with advanced cancer was correlated with high levels of ROS and low levels of antioxidant enzymes [27, 116, 117]. Oxidative stress can negatively regulate immune response; high ROS levels induce specific alterations in TCR signaling proteins, including conformational changes in TCRζ and LCK, thereby reducing their phosphorylation and signaling-induced calcium flux, with consequent suppression of antigen-mediated T-cell responses [118]. In particular, ovarian cancer ascites exhibit high levels of ROS and it has been demonstrated that incubation with the cell-free ascites enhances the ROS level in T cells as well as in dendritic cells, with consequent impairment of the antigen-presenting capacity of DCs and inhibition of T-cell activation [119]. In vitro treatment with antioxidants, such as α-lipoic acid and N-acetyl cysteine, reversed the oxidoreductive state and restored the blastic response of lymphocytes isolated from patients with advanced-stage cancer [120, 121]. These findings provided our group with the rationale for studies on the therapeutic effect of several antioxidants in patients with advanced cancer [122–124], and particularly of a combined regimen targeting symptoms associated with inflammation and oxidative stress in patients with advanced ovarian cancer [29]. Of relevance, Wu et al. [7] has reported the treatment of patients with severe COVID-19 with antioxidants like glutathione and N-acetyl cysteine. In this regard, we would like to mention our previous review on the role of carbocysteine, which focused primarily on the anti-inflammatory properties of such an irreplaceable antioxidant agent [125].

Moreover, ROS overproduction is sensed by the nucleotide-binding domain, leucine-rich-containing family, pyrin domain-containing-3 (NLRP3) inflammasome, an intracellular multiprotein sensor of danger signals induced by pathogens or cellular stress, which, upon activation, serves as a platform for activation of the cysteine protease and Caspase-1, which subsequently induces the production of pro-inflammatory cytokines, including IL-6. Further, ROS can directly oxidize thiol groups in the leucine-rich repeat domain of NLRP3 to activate the inflammasome pathway [126], which can further contribute to a prolonged, excessive inflammatory response in patients with COVID-19. This event may be common in elderly patients, the group with the highest COVID-19-related mortality rate, due to a phenomenon referred to as inflammaging.

Inflammaging, the low-grade pro-inflammatory status associated with aging [127], could contribute to explain the more severe effects of COVID-19 in the elderly population. Some studies have suggested that, in healthy elderly individuals, the circulating levels of inflammatory cytokines are higher than in younger individuals, even in the absence of a comorbid disease [128, 129]. The development of this chronic inflammatory status is attributed to activation of the inflammasome downstream of nuclear factor kappa-light-chain-enhancer of activated B cells (NF-κB) signaling, which become stimulated by cellular stresses that occur during the aging process. Similarly, an increased activation of inflammasome and its contribution to chronic inflammation has been demonstrated in malignant ovarian cancer [130]. Aging is characterized by ROS-mediated mitochondrial damage, an increase in iron deposition, deregulation of Ca^2+^ and postabsorptive plasma cysteine homeostasis, as well as impaired autophagy and sirtuin activity secondary to aberrant insulin receptor signaling; events that are observed also in advanced ovarian cancer [131–133]. These altered pathways influence progressive increases in intracellular ROS level and oxidative stress [134]. Moreover, the aging process involves a progressive decline in cellular and organism-level function; in particular, defects in mitochondrial degradation further increases ROS production. Thus, oxidative stress in elderly patients stimulates the NF-κB signaling via multiple mechanisms, while enhancing inflammasome activation [135, 136].

The consequent ROS-induced inflammasome activation has been hypothesized to be associated with the chronic inflammation, endothelial damage and to thrombotic events in the elderly. In particular, in vivo and in vitro studies have indicated that aging is associated with an increase in the steady-state levels of IL-6 mRNA resulting from enhanced binding of the redox-sensitive transcription factor NF-κB to the IL-6 promoter [137]. Consequently, older subjects present heightened basal levels of pro-inflammatory mediators, characterized primarily by mildly elevated levels of IL-6, which may contribute to their decreased pulmonary function and blunted immune responses to respiratory tract infections [138]. Thus, in the elderly, the inflammasome activation may further exacerbate the aspecific inflammatory response observed in COVID-19, making these patients more susceptible to the complications of this viral infection.

### COVID treatment strategy based on pathogenetic mechanisms

The chronicization of the hyperinflammatory cytokine storm observed in patients with severe COVID-19 highlights a defective resistance phase that leads to aspecific chronic inflammation responsible for the impairment of an effective specific T-cell response and subsequent deterioration of patients’ condition (tolerance phase), with systemic effects that can lead to severe multi-organ failure and death.

Thus, in the tolerance phase, targeting the symptoms consequent to chronic inflammation, such as thrombosis, anemia, anorexia, weight loss, muscle wasting is fundamental for an effective treatment strategy. Therefore, treatment with immunomodulatory and immunosuppressive drugs, such as glucocorticoids and low molecular weight heparin, to prevent or treat the associated thrombotic events, as well as administration of supportive drugs, such as antioxidants and lactoferrin, which help to restore the defective function of the adaptive specific immunity and modulate the altered iron metabolism, as observed in patients with advanced ovarian cancer [29, 139], may prove beneficial even in the absence of specific antiviral treatments. In a randomized clinical trial [140] we have demonstrated in patients with advanced cancer, primarily ovarian cancer, with chemotherapy-related anemia, that lactoferrin mobilized iron storage thereby reducing ferritin levels and increasing iron availability for erythropoiesis and heme proteins synthesis, such as myoglobin and cytochrome chain.

Indeed, corticosteroids significantly reduce mortality of critically ill COVID-19 patients [141]. In detail, the Randomized Evaluation of COVID-19 Therapy (RECOVERY) trial, a multicenter, randomized, open-label trial in hospitalized patients with COVID-19, showed that the mortality from COVID-19 was lower among patients who received dexamethasone than among those received standard care [142]. Also, a meta-analysis of seven randomized controlled trials including approximately 1700 patients with COVID-19 showed that systemic corticosteroids decrease 28-day mortality rate without safety concerns [143].

Furthermore, targeted drugs such anti-IL-6 monoclonal antibody (mAb), anti-IL-1 mAb, anti-TNF mAb, may counteract the cytokine storm syndrome. However, to date, results from the main randomized clinical trials on hospitalized patients with COVID-19 treated with the anti-IL-6 mAb, tocilizumab, remain controversial [144–147]*.* Notably, the early use in severe patients has been reported to be associated with lower progression to mechanical ventilation and mortality [141, 142]*.* Further studies are ongoing [148]; while another anti-IL-6 mAb, siltuximab, is currently being tested in several ongoing randomized clinical trials [149].

Additionally, JAK inhibitors have been suggested as a promising potential agent against COVID-19 owing to their ability to inhibit both the JAK/STAT pathway, with its associated cytokine signaling, and AP2-associated protein kinase 1, which regulates SARS-CoV-2 endocytosis via the ACE receptor expressed on epithelial alveolar lung cells [22, 150].

Convalescent plasma is potentially effective against COVID-19, however, to date, only data from retrospective case series are available and adequately powered, randomized controlled trials remain warranted [151]. Similarly, anti-SARS-CoV-2 neutralizing mAbs (or mAb cocktails) are also under investigation in ongoing Phase 2 or Phase 3 clinical trials [152].

Undoubtedly, the gold standard is the prevention with vaccines and the use of antiviral therapy, particularly in the early stages of the disease (resistance phase); to this regard, several trials testing potential antiviral agents are underway [153]. Among them the most promising agent seems remdesivir; in a randomized clinical trial, compared with placebo, remdesivir shortened the time to recovery in adults hospitalized with COVID-19 and lowered respiratory tract infection [154]. Co-administration of antiviral agents with immunosuppressive/anti-inflammatory drugs, such as corticosteroids and JAK inhibitors are being evaluated [155].

As for SARS-CoV-2 vaccine, the preliminary results with RNA-based vaccines were positive. In particular, an ongoing multinational, placebo-controlled, observer-blinded, pivotal efficacy trial, tested the efficacy and safety of the vaccine with BNT162b2, a lipid nanoparticle–formulated, nucleoside-modified RNA vaccine that encodes a prefusion stabilized, membrane-anchored SARS-CoV-2 full-length spike protein [156]. In the primary analysis, which included approximately 20,000 subjects for each arm, BNT162b2 was 95% effective in preventing COVID-19 (95% credible interval, 90.3 to 97.6): Only eight cases were observed in the vaccine group as compared with the 162 in the placebo group. Among 10 cases of severe COVID-19 with onset after the first dose, nine occurred in placebo recipients and one in a BNT162b2 recipient. The safety profile of BNT162b2 was characterized by short-term, mild-to-moderate pain at the injection site, fatigue, and headache. The incidence of serious adverse events was low and similar both groups [156]. Another randomized, placebo-controlled study tested the mRNA-1273 vaccine, with 30,000 adults receiving two 100 μg doses 1 month apart. The interim data from the trial based on 95 cases estimated efficacy at 94.5%; moreover, all severe cases as well as one COVID-19 related death occurred in the placebo group. At interim analysis, no serious adverse events occurred [157]. These results led to emergency authorization from the US Food and Drug Administration and conditional approval from the European Medicines Agency for these COVID-19 vaccines. More recently, the viral vector vaccine ChAdOx1 nCoV-19 showed an acceptable safety profile and has been found to be efficacious (an overall vaccine efficacy of 70.4%) against symptomatic COVID-19 in an interim analysis of ongoing clinical trials including 11,683 participants [158].

Therefore, it is essential to define the clinical conditions of each patient with COVID-19 and identify the most appropriate treatment for each disease stage based on laboratory data essential to define the evolution of the infection [7, 9, 22, 23]. In patients where the presence of few symptoms is prevalent (pauci-symptomatic subjects), the use of paracetamol, aspirin, and other cyclooxygenase-2 (COX2) inhibitors, appears to be more appropriate [159]. The role of aspirin is being investigated in patients with COVID-19 in RECOVERY trial [160]. Notably, in other SARS coronaviruses, COX-2 pathway could be directly activated by the virus, thus boosting prostaglandin production, and is involved in mediating viral RNA synthesis and replication as well as influencing antiviral lymphocyte response [161]. In patients with moderately symptomatic (mild severe) disease and altered inflammatory indices, the use of low-dose glucocorticoids and low molecular weight heparin at prophylactic dosage calculated based on body weight, rather than the standard dose of 4000 IU often used in clinical practice, should be used. Meanwhile, in severe and terminal stages, the use of high dose glucocorticoids, low molecular weight heparin (based on body weight), and antithrombin III, with or without the administration of selective cytokines inhibitors, is the most appropriate therapeutic approaches. Additionally, throughout the course of the disease, regardless of severity, the use of antioxidants must be included, such as acetylcysteine ​​or carbocysteine which are readily available.

## Conclusion

In conclusion, to facilitate administration of the most effective treatments, it is essential to correctly identify the stages of COVID-19 infection, and to select an appropriate and specific therapeutic protocol based on the most available data. As outlined in other disease models with similar immunopathology including ovarian cancer, severe COVID-19 therapies must target inflammation, oxidative stress, coagulation disorders, and nutritional status to improve patient outcomes. Recently, we have highlighted the fundamental need to modulate inflammation and related disorders to improve the outcomes of immunotherapies in patients with advanced cancer [162]. Hence, the potential role of checkpoint inhibitors, such as the programmed death (PD)-1 ligand, which its inhibition restores the function of exhausted lymphocytes, are being examined in these patients which could provide additional therapeutic perspectives [163].

## Abbreviations

SARS-CoV-2: Severe acute respiratory syndrome coronavirus 2
COVID-19: Corona Virus Disease 2019;
ARDS: Acute respiratory distress syndrome
CRP: C-reactive protein
NLR: Neutrophil/lymphocyte ratio
IL: Interleukin
TNF: Tumor necrosis factor
ROS: Reactive oxygen species
IFNs: Interferons
ACE2: Angiotensin-converting enzyme 2
HIF: Hypoxia-inducible factor
STAT3: Signal transducer and activator of transcription 3
PI3K: Phosphatidylinositol-3-kinase
AKT: Protein kinase B
mTOR: Mammalian target of rapamycin
JAK: Janus kinase
SH2: Src Homology 2
TCR: T-cell receptor
NLRP3: Nucleotide-binding domain, leucine-rich-containing family, pyrin domain-containing-3
NF-κB: Nuclear factor kappa-light-chain-enhancer of activated B cells
mAb: Monoclonal antibody
AP: Adaptor protein
COX2: Cyclooxigenase-2
PD: Programmed death
